# Genome-Wide Identification and Functional Characterization of CCHC-Type Zinc Finger Genes in *Ustilaginoidea virens*

**DOI:** 10.3390/jof7110947

**Published:** 2021-11-10

**Authors:** Xiaoyang Chen, Zhangxin Pei, Lin Peng, Qin Qin, Yuhang Duan, Hao Liu, Xiaolin Chen, Lu Zheng, Chaoxi Luo, Junbin Huang

**Affiliations:** 1 Hubei Key Laboratory of Plant Pathology, Huazhong Agricultural University, Wuhan 430070, China; chenxiaoyang2345@163.com (X.C.); Plin18943606383@163.com (L.P.); hzauqinqin980223@163.com (Q.Q.); duan199607272021@163.com (Y.D.); hl210@mail.hzau.edu.cn (H.L.); chenxiaolin@mail.hzau.edu.cn (X.C.); luzheng@mail.hzau.edu.cn (L.Z.); cxluo@mail.hzau.edu.cn (C.L.); 2 State Key Laboratory of Agricultural Microbiology, Huazhong Agricultural University, Wuhan 430070, China; 3 Wuhan Institute of Landscape Architecture, Wuhan 430081, China; peizhangxinhzau@163.com

**Keywords:** rice false smut, *Ustilaginoidea virens*, CCHC-type zinc finger proteins, development, virulence

## Abstract

Rice false smut caused by *Ustilaginoidea virens* is a serious disease of rice (*Oryza sativa*), severely reducing plant mass and yields worldwide. We performed genome-wide analysis of the CCHC-type zinc-finger transcription factor family in this pathogen. We identified and functionally characterized seven *UvCCHC* genes in *U. virens*. The deletion of various *UvCCHC* genes affected the stress responses, vegetative growth, conidiation, and virulence of *U. virens*. ∆*UvCCHC5* mutants infected rice spikelets normally but could not form smut balls. Sugar utilization experiments showed that the ∆*UvCCHC5* mutants were defective in the utilization of glucose, sucrose, lactose, stachyose, and trehalose. Deletion of *UvCCHC5* did not affect the expression of rice genes associated with grain filling, as revealed by RT-qPCR. We propose that the ∆*UvCCHC5* mutants are impaired in transmembrane transport, and the resulting nutrient deficiencies prevent them from using nutrients from rice to form smut balls. RNA-seq data analysis indicated that *UvCCHC4* affects the expression of genes involved in mitochondrial biogenesis, ribosomes, transporters, and ribosome biogenesis. These findings improve our understanding of the molecular mechanism underlying smut ball formation in rice by *U. virens*.

## 1. Introduction

Transcription factors are essential players in the development and virulence of plant pathogens that directly or indirectly regulate the expression of downstream genes [1]. The transcription factor families involved in phytopathogen development and virulence include bZIP (basic leucine zipper), bHLH (basic helix-loop-helix), homeodomain-like, MYB, MADS-box, APSES, and zinc-finger proteins [2]. The zinc-finger protein family is a large and diverse superfamily of proteins. Zinc-finger proteins use finger-like structures containing Zn^2+^ to bind DNA or RNA nucleotides and recognize DNA–RNA complexes or other proteins [3], thereby regulating the transcription or translation of genes that function in crucial processes such as growth, apoptosis, cell proliferation, and differentiation. The zinc finger is a tetrahedral structure formed by cysteine (Cys) and histidine (His) combined with zinc ions. Zinc-finger proteins are divided into different types based on their number and arrangement of Cys and His residues, such as C2HC, C2H2, C3HC4, C2HC5, and so on [4], as well as CCHC-type zinc finger proteins.

CCHC-type zinc-finger proteins are highly conserved and widely distributed in animals, plants, and microorganisms [5]. All CCHC-type zinc-finger proteins contain the conserved CCHC-box domain “-C-X2-C-X4-H-X4-C-”. The number or spatial structure of the CCHC box determines the type of motif or protein that it can recognize. In general, zinc-finger proteins containing one CCHC box bind to single-stranded DNA or RNA. Zinc-finger proteins containing three CCHC boxes are better able to bind to single-stranded RNA, including mRNA or miRNA in eukaryotic cells [6,7]. In plants, CCHC-type zinc-finger proteins play key roles in regulating growth, development, and both abiotic and biotic stress responses [8,9,10,11,12,13,14,15,16,17,18,19]. In animals, these proteins are involved in regulating DNA recognition, RNA processing, transcriptional activation, cell apoptosis, and so on [20,21,22]. The yeast genome encodes at least seven CCHC-type zinc-finger proteins including Sf1, Slu7, Mpe1, Air1, Air2, Gis2, and Bik1; six of these are involved in RNA metabolism [5]. Sf1 is involved in the assembly of early spliceosome complexes. Slu7 is required for splicing fidelity [23,24]. Mpe1 is required for pre-mRNA cleavage and for polyadenylation at the *bona fide* site [25]. Air1 and Air2 are RNA-binding proteins [5]. Gis2 is considered a translational activator since it promotes cap-independent translation through interactions with the 5′-terminal oligopyrimidine tract of mRNAs and the translating ribosome [26]. Bik1 plays critical roles in the regulation of microtubule polymerization, stabilization, and dynamics [27]. However, the roles of CCHC-type zinc-finger proteins in fungal pathogens are still unknown.

Rice false smut caused by *Ustilaginoidea virens*, a devastating grain disease of rice, has become a problem in most rice-growing areas worldwide [28]. In addition to causing yield losses, rice false smut also threatens human and animal health by producing cyclopeptide mycotoxins in smut balls [29,30]. Comparative genomic analysis of *U. virens* and other filamentous ascomycetes predicted that *U. virens* possesses over 300 transcription factors [31,32], but only a few have thus far been characterized. For example, UvPRO1 plays key roles in the stress responses, vegetative growth, conidiation, and virulence of *U. virens* [33]. UvHox2 regulates the conidiation, chlamydospore formation, and virulence of *U. virens* [34]. UvCom1 regulates the conidiation, stress responses, vegetative growth, and virulence of *U. virens* [35]. The Zn(2)-Cys(6) class fungus-specific transcription factor UvZnFTF1 is involved in the conidiation, vegetative growth, pigment biosynthesis, and virulence of *U. virens* [36]. The C2H2-type zinc-finger transcription factor UvMSN2 plays a key role in the conidiation, stress responses, vegetative growth, mitochondrial morphology, and virulence of *U. virens* [37]. The C2H2-type zinc-finger transcription factor UvCGBP1 also plays important roles in the conidiation, stress responses, vegetative growth, and virulence of *U. virens*. Finally, UvCGBP1 regulates the translation and transcription of the MAPK pathway kinase gene *UvSlt2*, which plays an important role in virulence [38]. Nonetheless, the roles of most transcription factors in *U. virens* are unknown.

In this study, we performed genome-wide identification and functional characterization of CCHC-type zinc-finger proteins in *U. virens*. Our findings demonstrate that CCHC-type zinc-finger proteins play important roles in regulating smut ball formation, conidiation, stress responses, vegetative growth, and virulence in *U. virens*.

## 2. Materials and Methods

### 2.1. Fungal Strains and Growth Conditions

The *U. virens* wild-type strain HWD-2 and all transformed strains were cultured on potato sucrose agar (PSA) medium at 28 °C in the dark. Seven-day-old mycelia cultured in potato sucrose broth (PSB) shaken at 180 rpm were used to isolate fungal DNA/RNA, and conidia separated from the culture were used for *Agrobacterium tumefaciens*-mediated transformation (ATMT) as described by Chen et al. [39].

### 2.2. Transactivation Activity Assay in Yeast

The transcriptional activities of UvCCHC proteins were examined by yeast one-hybrid assays using the Matchmaker pGBKT7 (Clontech, Mountain View, CA, USA) system. The coding sequences of *UvCCHC* genes were ligated into the pGBKT7 vector for expression under the control of the GAL4 promoter. The resulting pGBKT7-*UvCCHC* vectors were transformed into Y1HGold (Clontech, Mountain View, CA, USA) cells, and transformants were isolated in SD/–Trp medium and confirmed by PCR with primer pair P1/P2. The transcriptional activities of the UvCCHC proteins were examined based on yeast growth on SD/–Trp/–His medium with X-α-Gal (Clontech, Mountain View, CA, USA).

### 2.3. DNA/RNA Manipulation and RT-qPCR

Genomic DNA was extracted from vegetative hyphae using the CTAB method. Total RNA was extracted from vegetative hyphae and infected rice spikelets (1, 3, 5, 7, 9, or 13 days) using an RNA kit (Thermo Fisher Scientific, Waltham, MA, USA). cDNA synthesis was performed using cDNA Synthesis SuperMix (TransGen Biotech, Beijing, China). Quantitative reverse-transcription PCR (RT-qPCR) was conducted using TransStart^®^ Tip Green qPCR SuperMix (TransGen Biotech, Beijing, China) to detect the expression levels of *UvCCHC* or rice grain filling–associated genes at different stages of infection with primer pairs (Supplementary Table S1); the *U. virens* β-tubulin (*Uv8b_900*) or rice ubiquitin gene (*OsUBQ1*), respectively, were used as internal controls [40].

### 2.4. Gene Deletion and Complementation

Deletion mutants of *UvCCHC* genes were generated using ATMT. Approximately 1000 bp of the downstream and upstream flanking sequences of the genes were ligated into the pGKO deletion vector. The pGKO-*UvCCHC* constructs were inserted into *A. tumefaciens* strain EHA105 cells, which were used to transform HWD-2 conidia. Hygromycin-resistant transformants were isolated and confirmed by PCR. For the complementation assays, an approximately 5-kb fragment containing a 1.5-kb native promoter region and the full-length *UvCCHC* gene sequence were amplified with primer pair P7/P8, and the resulting PCR products were ligated into pNeo3300III. *A. tumefaciens* strain EHA105 harboring the pNeo3300III-*UvCCHC* constructs were transformed by ATMT by co-culturing the agrobacteria with conidia of the Δ*UvCCHC* mutants. G418-resistant transformants (those carrying constructs) were isolated and confirmed by PCR with primer pairs P1/P2, P3/P4, P3/P6 and P5/P6.

### 2.5. Vegetative Growth and Conidiation

For vegetative growth, 5-mm mycelial plugs were obtained from 10-d-old PSA plates and grown on fresh PSA medium at 28 °C. After 14 d of incubation, the radial growth of vegetative mycelia was measured. For conidial production, strains were grown in PSB medium at 28 °C. After shaking at 180 rpm for 7 d, the cultures were filtered through four layers of gauze, and conidial production was measured using a hemocytometer. Each treatment was repeated three times [39].

### 2.6. Stress Adaptation Assays and Sugar Utilization Assays

To test the sensitivity of the cultures to environmental stress, 5-mm mycelial plugs were cultured on PSA plates containing 0.25 M NaCl, 0.5 M sorbitol, 0.02% or 0.04% H_2_O_2_, 0.03% sodium dodecyl sulfate (SDS), 120 μg/mL Calcofluor white (CFW), or 120 μg/mL Congo red (CR). For the sugar utilization assays, after 10 d of growth on PSA plates, 5-mm mycelial plugs of HWD-2 and the mutated strains ∆*UvCCHC5-23*, ∆*UvCCHC5-43*, and C∆*UvCCHC5-23* were transferred to Czapek-Dox Agar medium (2 g KNO_3_, 1 g K_2_HPO_4_, 0.01 g FeSO_4_, 0.5 g KCl, 0.5 g MgSO4·7H_2_O, 16 g agar, 30 g sugar sources, with ddH_2_O to a total volume of 1000 mL) with different sugar sources, including glucose, sucrose, lactose, maltose, raffinose, stachyose, trehalose, and soluble starch. After 14 d of incubation, the radial growth of vegetative mycelia was measured; three replicates were grown per strain.

### 2.7. Pathogenicity and Plant Infection Assays

The susceptible rice cultivar Wanxian-98 was used for pathogenicity assays [36]. The wild-type HWD-2, ∆*UvCCHC* mutant, and complementation strains were cultured in PSB medium at 28 °C for 7 d with shaking at 180 rpm. Rice plants were inoculated with 2 mL mycelial/spore suspensions (1 × 10^6^ conidia/mL) at the booting stage using a syringe. The inoculated rice plants were cultivated in a greenhouse at 25 °C with a relative humidity of 100% for 7 d, and then grown in a greenhouse at 28 °C with a relative humidity of 90% for a further 14 d. The number of smut balls was then counted. The inoculation experiment was repeated three times, and each strain was inoculated onto 15 panicles each time. Rice spikelet samples were collected at three days post-inoculation (dpi) and 5 dpi in 2.5% glutaraldehyde fixative. The samples were critical-point dried, mounted on stubs, sputter coated with gold-palladium, and viewed under a JEOL JSM-6390LV scanning electron microscope (SEM) operating at 10 kV.

### 2.8. Generation of the GFP-UvCCHC Fusion Construct

The full-length *UvCCHC4* or *UvCCHC5* coding sequence was ligated into the pNeo3300III-GFP vector. The pNeo3300III-GFP-*UvCCHC4* and pNeo3300III-GFP-*UvCCHC5* constructs were inserted into *A. tumefaciens* strain EHA105 cells, which were used to transform Δ*UvCCHC4-1* and Δ*UvCCHC5-23* conidia, respectively. The transformants were verified by PCR with primer pair P1/P2 and immunoblotting. GFP-tagged strains were chosen to observe the subcellular localizations of the resulting fusion proteins under a Zeiss LSM510 Meta confocal microscope (Zeiss, Jena, Germany).

### 2.9. RNA-seq and Data Analysis

Total RNA extracted at 3 dpi from three biological replicates of rice spikelets infected with the HWD-2 and Δ*UvCCHC4-1* strains was used for RNA sequencing (RNA-seq). RNA-seq libraries were constructed using an NEBNext^®^ Ultra^™^ Directional RNA Library Prep Kit using 5 μg total RNA. RNA was first isolated using the polyA selection method by oligo(dT) beads and then fragmented by fragmentation buffer. Double-stranded cDNA was then synthesized using a SuperScript double-stranded cDNA Synthesis Kit (Invitrogen, Carlsbad, CA, USA). Then the synthesized cDNA was subjected to end-repair, phosphorylation and ‘A’ base addition according to Illumina’s library construction protocol. Libraries were size-selected for cDNA target fragments of 200–300 bp on 2% Low Range Ultra Agarose followed by PCR. After quantification by TBS380, paired-end RNA-seq libraries were sequenced on an Illumina HiSeq 4000 system with 150-bp paired-end reads at IGENEBOOK Biotechnology Co., Ltd. (Wuhan, China). For RNA-seq data analysis, the raw data of paired-end reads were filtered using SeqPrep and Sickle with default parameters. Cufflinks (version 2.2.1) and TopHat (version 2.0.14) software were used to map clean reads to the *U. virens* genome and to calculate differential expression. The expression level of each transcript was calculated as fragments per kilobase of exon per million mapped reads (FRKM). RSEM software (http://deweylab.biostat.wisc.edu/rsem/, accessed on 24 May 2021) was used to quantify gene abundances [41]. The filter conditions of fold change >2 and adjusted *p* <  0.05 were applied to identify differentially expressed genes. Gene Ontology (GO) functional enrichment and Kyoto Encyclopedia of Genes and Genomes (KEGG) pathway analysis were carried out by Goatools (https://github.com/tanghaibao/Goatools, accessed on 20 July 2021) and KOBAS (http://kobas.cbi.pku.edu.cn/home.do, accessed on 22 July 2021) [42].

### 2.10. Accession Numbers

The accession numbers of the rice grain filling–associated genes mentioned in this article are: *OsSSIIIa* (LOC_Os08g09230); *OsRISBZ1* (LOC_Os07g08420); *OsBEIIb* (LOC_Os02g32660); *OsAGPS2b* (LOC_Os08g25734); *OsAGPL2* (LOC_Os01g44220); *OsPromln2* (LOC_Os05g26770)*; OsSSI* (LOC_Os06g06560); *OsGlutln3* (LOC_Os02g15090); *OsUBQ1* (LOC_Os03g13170).

### 2.11. Statistical Analysis

The data were statistically analyzed using SPSS version 16.0 software (SPSS Inc., Chicago, IL, USA) and are presented as the mean ± standard deviation (SD).

## 3. Results

### 3.1. Identification of CCHC-Type Zinc-Finger Proteins in U. virens

**Figure 1:**
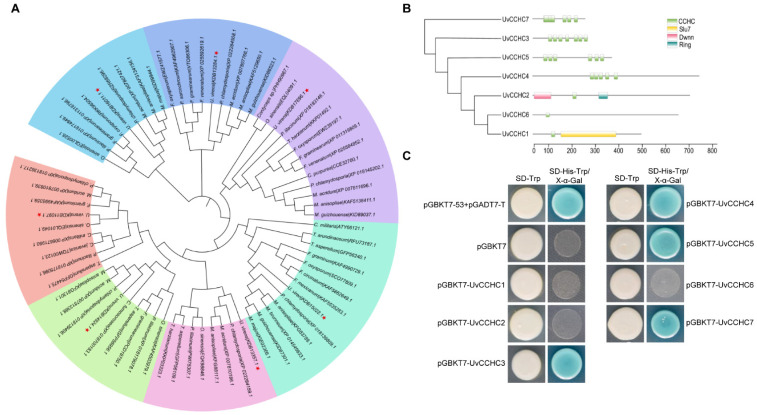
Identification of CCHC-type zinc-finger proteins in *U. virens*. (**A**) Neighbor-joining tree of CCHC-type zinc-finger protein homologs from different fungal genomes generated with MEGA7.0. The bootstrap percentage values from 1000 repeats are shown at the branch nodes. (**B**) Predicted Pfam domains of CCHC-type zinc-finger (box) proteins. (**C**) Transactivation analysis of CCHC-type zinc-finger proteins in yeast. The vectors pGBKT7-53/pGADT7-T and pGBKT7 were expressed in yeast as positive and negative controls, respectively.

We identified seven CCHC-type zinc-finger genes in the *U. virens* genome based on the presence of the conserved CCHC-box domain “-C-X2-C-X4-H-X4-C-”. Phylogenetic analysis of CCHC-type zinc-finger protein homologs from different fungi revealed that these proteins are conserved in filamentous fungi (Figure 1A). We identified seven genes encoding proteins containing one to seven copies of the conserved CCHC box, suggesting that seven CCHC-type zinc-finger proteins are present in this fungus (Figure 1B; Table 1). To explore the transcriptional activity of these proteins, we performed a yeast one-hybrid assay by transforming Y1HGold cells with pGBKT7-*UvCCHC* constructs harboring the seven putative *UvCCHC* genes. Transformants harboring *UvCCHC3, UvCCHC4*, *UvCCHC5*, or *UvCCHC7* grew normally and appeared blue on SD/–Trp/–His plates, revealing transactivation activity in yeast (Figure 1C). These results suggest that UvCCHC3, UvCCHC4, UvCCHC5, and UvCCHC7 function as transcription factors in *U. virens*.

### 3.2. Expression Patterns of CCHC-Type Zinc-Finger Genes in U. virens

**Figure 2:**
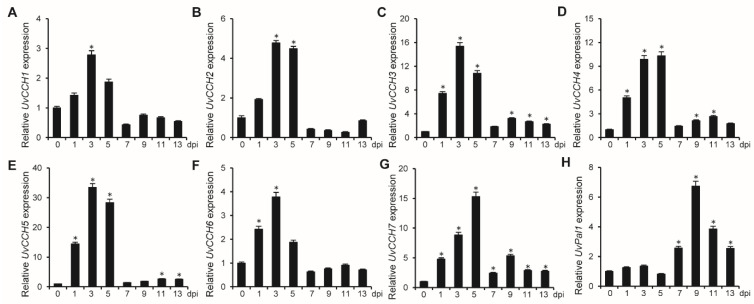
Expression of *UvCCHC* genes in *U. virens*. (**A**–**H**) Expression profiles of *UvCCHC* and *UvPal1* genes relative to *β-tubulin* in hyphae (0 dpi) in PSB and at different stages of infection on rice (1–13 d), as determined by RT-qPCR. Asterisks represent significant differences in comparison with 0 dpi at *P* = 0.05.

To further investigate the functions of the UvCCHC transcription factors*,* we examined the expression profiles of these *UvCCHC* genes in *U. virens* at various stages of infection through RT-qPCR with primer pair P9/P10. Compared to the vegetative-mycelial stage (0 dpi), *UvPal1* was highly expressed during mycelial expansion to form smut balls (7–13 dpi) in rice spikelets, as a control [39], and the expression levels of *UvCCHC* genes significantly increased during the early stage of hypha infection in rice floral organs at 3–5 dpi (Figure 2). These observations suggest that *UvCCHC* genes play important roles during early infection of *U. virens*.

### 3.3. UvCCHC Genes Are Important for the Mycelial Growth and Conidiation of U. virens

**Figure 3:**
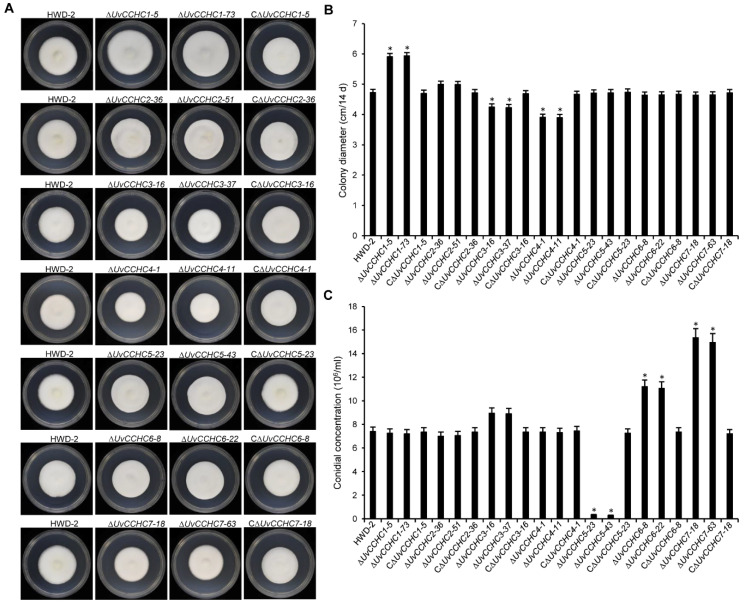
Deletion of *UvCCHC* genes affects hyphal growth and conidiation. (**A**) Colony morphology of HWD-2, Δ*UvCCHC* mutants, and complementation strains grown on PSA for 14 d. (**B**) Colony diameter of the mutant strains grown on PSA for 14 d. (**C**) Conidial production of the mutant strains incubated in PSB at 180 rpm for 7 d. Asterisks represent significant differences between HWD-2 and the mutants at *p* = 0.05, as determined by least significant difference (LSD) test.

We generated *UvCCHC* gene knockout mutants by deleting the *UvCCHC* genes using a homologous recombination strategy, as confirmed by PCR analysis (Supplementary Figure S1; Table 2). To investigate whether *UvCCHC* genes are associated with the mycelial growth of *U. virens*, we measured the growth rates of wild-type strain HWD-2*,* the ∆*UvCCHC* mutants, and the complementation strains on PSA medium. Compared to that of HWD-2, the mycelial growth rates of the ∆*UvCCHC3* and ∆*UvCCHC4* mutants were significantly reduced, whereas those of the ∆*UvCCHC1* mutants were significantly increased (Figure 3A,B). We also assessed conidiation in all *U. virens* strains when incubated in PSB with shaking. Conidial production was significantly reduced in the ∆*UvCCH5* mutant but significantly increased in the ∆*UvCCH6* and ∆*UvCCH7* mutants compared to wild-type HWD-2 (Figure 3C). These results suggest that *UvCCHC1*, *UvCCHC3*, and *UvCCHC4* are required for mycelial growth and that *UvCCHC5*, *UvCCHC6*, and *UvCCHC7* play important roles in the conidiation of *U. virens.*

### 3.4. UvCCHC Genes Play Key Roles in the Responses of U. virens to Environmental Stress

**Figure 4:**
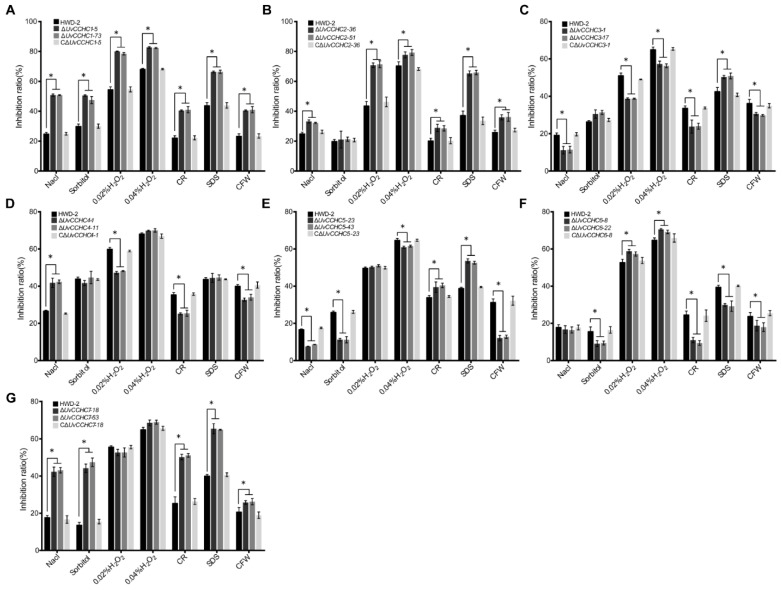
The ∆*UvCCHC* mutants are sensitive to various stresses. (**A**–**G**) Inhibition of colony growth of the mutant strains on PSA supplied with 0.25 M NaCl, 0.5 M sorbitol, 0.02% or 0.04% H_2_O_2_, 0.03% SDS, 0.12 mg/mL CR, or 0.12 mg/mL CFW after 14 d of incubation at 28 °C. Asterisks represent significant differences between HWD-2 and the mutants at *p* = 0.05, as determined by LSD test.

To explore the roles of *UvCCHC* genes in mediating the adaptation of *U. virens* to environmental stress, we compared the radial growth rates of HWD-2, the ∆*UvCCHC* mutants, and the complementation strains on PSA containing different stress agents. These chemicals significantly inhibited the mycelial growth of the ∆*UvCCHC* mutants compared to HWD-2 (Figure 4; Supplementary Figure S2). These results indicate that *UvCCHC* genes regulate the responses of *U. virens* to osmotic stress and oxidative stress, as well as its cell wall integrity.

### 3.5. UvCCHC Genes Play Crucial Roles in the Virulence of U. virens

**Figure 5:**
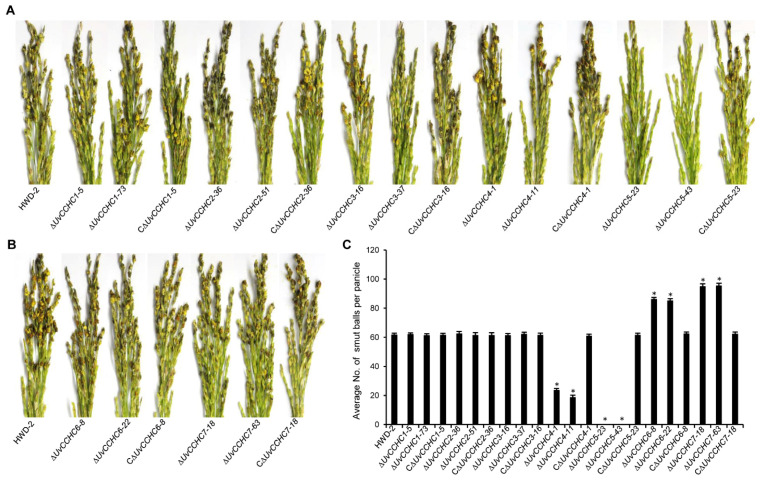
*UvCCHC* genes are important for the virulence of *U. virens*. (**A**,**B**) Virulence assays of HWD-2, the Δ*UvCCHC* mutants, and complementation strains on rice spikelets at 21 dpi. (**C**) Average number of smut balls per panicle. Asterisks represent significant differences relative to HWD-2, as determined by LSD test at *p* = 0.05.

To explore the roles of the *UvCCHC* genes in *U. virens* infection, we conducted virulence assays of HWD-2, the ∆*UvCCHC* mutants, and the complementation strains on the susceptible rice cultivar Wanxian-98. After 21 dpi, we observed approximately 62 smut balls on rice spikelets infected by HWD-2 and the complementation strains, and approximately 60 smut balls on rice spikelets infected by ∆*UvCCHC1,* ∆*UvCCHC2*, and ∆*UvCCHC3.* The pathogenicity of ∆*UvCCHC6* and ∆*UvCCHC7* was significantly higher, as rice spikelets infected by these mutants produced more than 90 smut balls. By contrast, the virulence of the ∆*UvCCHC4* and ∆*UvCCHC5* mutants was significantly reduced, as ∆*UvCCHC4* produced only approximately 24 smut balls on rice spikelets (Figure 5), and the ∆*UvCCHC5* mutants failed to produce smut balls. These results indicate that *UvCCHC6* and *UvCCHC7* negatively regulate the virulence of *U. virens*, *UvCCHC4* and *UvCCHC5* positively regulate the virulence of this pathogen, and *UvCCHC1, UvCCHC2*, and *UvCCHC3* have no effect on its virulence.

### 3.6. UvCCHC5 Regulates the Formation of Smut Balls

**Figure 6:**
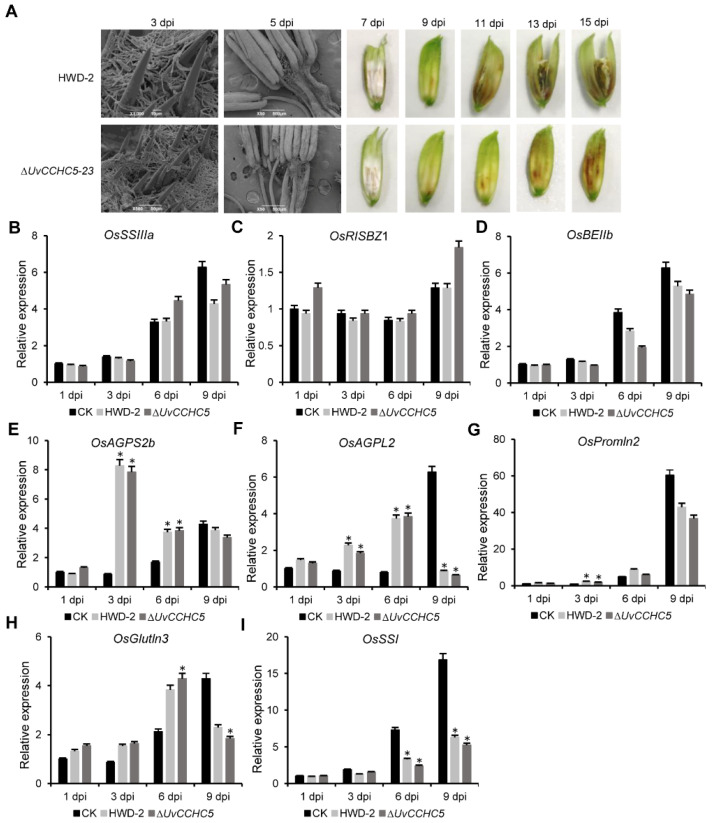
UvCCHC5 is a key transcription factor governing smut ball formation in rice. (**A**) Process of infection in inoculated rice spikelets. (**B**–**I**) Expression of genes associated with grain filling in rice at different stages after inoculation with ∆*UvCCHC5-23* and HWD-2 (1, 3, 6, and 9 d), as determined by RT-qPCR. Error bars represent the standard deviation of three replicates in independent experiments. Asterisks represent significant differences in comparison with CK at *P* = 0.05.

Although the ∆*UvCCHC5* mutants failed to produce smut balls, these mutants were able to infect rice floral organs. Therefore, we observed the infection process of HWD-2 and ∆*UvCCHC5-23* in more detail (Figure 6A). In both strains, at 3 dpi, hyphae elongated and extended along the surfaces of spikelets. At 5 dpi, hyphae were observed on the surfaces of filaments in colonized rice. At 15 dpi, rice grains infected by ∆*UvCCHC5*-*23* did not contain ball-like colonies, whereas infection by HWD-2 led to the formation of smut balls (Figure 6A). These results indicate that ∆*UvCCHC5*-*23* successfully established a nutritional relationship with rice spikelets but could not form smut balls.

### 3.7. Deletion of UvCCHC5 Does Not Affect the Expression of Rice Genes Associated with Grain Filling

*U. virens* infection in rice induces the expression of genes associated with grain filling by simulating fertilization, causing the rice to provide large amounts of nutrients to the pathogen [43,44]. We reasoned that unlike wild-type HWD-2, the ∆*UvCCHC5-23* mutant cannot induce the expression of grain-filling genes and therefore cannot obtain large amounts of nutrients, preventing the formation of smut balls. To explore this hypothesis, we measured the expression of genes associated with grain filling in rice after inoculation with ∆*UvCCHC5-23* or HWD-2 at different stages of infection. The expression levels of rice grain filling–associated genes, including *OsSSIIIa*, *OsRISBZ1*, *OsBEIIb*, *OsAGPS2b*, *OsSSI*, *OsPromln2, OsAGPL2*, and *OsGlutln3*, were similar in rice spikelets inoculated with the ∆*UvCCHC5-23* mutant and HWD-2 (Figure 6B–I). These results indicate that the deletion of *UvCCHC5* did not affect the expression of rice genes associated with grain filling. Therefore, the failure of ∆*UvCCHC5*-*23* to induce smut ball formation is likely due to a deficiency in pathogen development.

### 3.8. UvCCHC5 Plays Important Roles in Sugar Utilization, Carbohydrate Transport, and Transmembrane Transport

**Figure 7:**
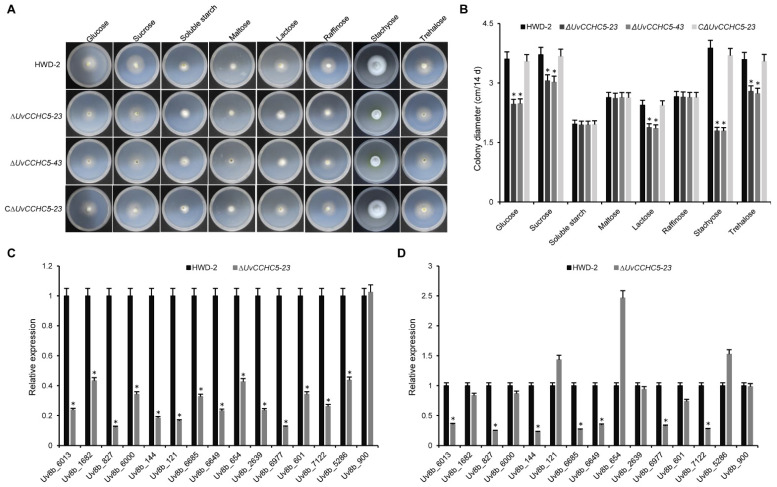
∆*UvCCHC5* mutants have defects in the utilization of different saccharides. (**A**) Colony morphology of ∆*UvCCHC5* mutant and complementation strains at 14 d of culture on plates containing different saccharides. (**B**) Colony diameter of ∆*UvCCHC5* mutants at 14 d of culture on plates containing different saccharides. (**C**,**D**) Results of RT-qPCR to validate the expression of 14 differentially expressed genes related to transmembrane transporters of *U. virens* in rice inoculated with HWD-2 and ∆*UvCCHC5-23* in samples obtained at 0 (**C**) and 11 (**D**) dpi. Asterisks represent significant differences relative to HWD-2, as determined by LSD test at *p* = 0.05.

Like wild-type *U. virens*, ∆*UvCCHC5*-*23* induced the expression of genes associated with grain filling in rice spikelets. We reasoned that the failure of infected spikelets to form smut balls was due not to the lack of a nutrient supply but instead to the impaired use of nutrients by *U. virens* after *UvCCHC5* gene knockout. Therefore, we compared the carbohydrate utilization preferences of ∆*UvCCHC5-23* and ∆*UvCCHC5-43* with those of HWD-2 and the complementation strain C∆*UvCCHC5-23*. We grew 5-mm mycelial plugs from these strains on Czapek-Dox Agar medium containing different saccharides, including sucrose, lactose, glucose, maltose, stachyose, raffinose, trehalose, and soluble starch. Compared to HWD-2 and C∆*UvCCHC5-23*, the growth rates of the ∆*UvCCHC5* mutants were significantly reduced on glucose, sucrose, lactose, stachyose, and trehalose media, indicating that the mutant had defects in the utilization of these saccharides (Figure 7A,B). These results suggest that *UvCCHC5* is involved in the utilization of glucose, sucrose, lactose, stachyose, and trehalose by *U. virens*.

We previously demonstrated that the transcription factor UvCom1 plays a key role in governing smut ball formation [35]. Here, we investigated whether UvCCHC5 shares the same function as UvCom1. UvCom1 regulates the expression of genes related to transporter activity, transmembrane transport, carbohydrate transport, and metabolism. Therefore, we performed RT-qPCR to detect the expression levels of genes related to membrane transport and sugar transport in mycelia. We found that these genes were significantly downregulated in ∆*UvCCHC5-23* (Figure 7C). In rice spikelets at 11 d after *U. virens* infection, wild-type hyphae absorb large amounts of nutrients from rice to form smut balls. We therefore measured the expression levels of these genes at 11 dpi, finding that most genes were significantly downregulated in ∆*UvCCHC5-23* relative to the wild type at this stage (Figure 7D). These results suggest that *U. virens* with a deletion of *UvCCHC5* is unable to form smut balls since the fungus itself has problems transporting rice nutrients, preventing it from utilizing these nutrients to form smut balls.

### 3.9. Genome-Wide Identification of Genes Regulated by UvCCHC4

**Figure 8:**
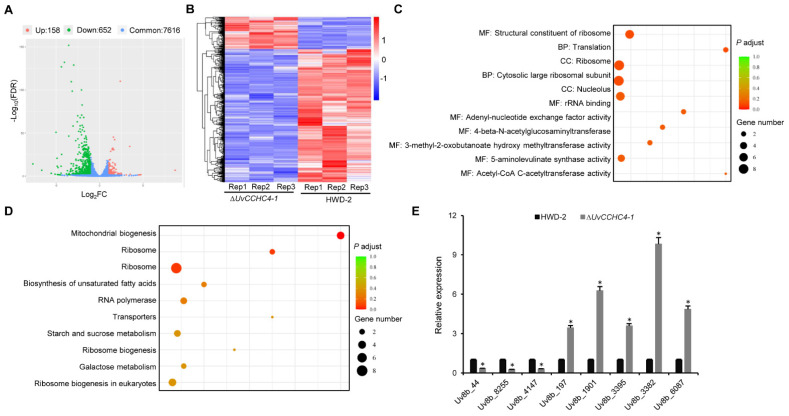
Transcriptomic analysis to identify DEGs in ∆*UvCCHC4-1* vs. HWD-2. (**A**) Volcano plot of DEGs in ∆*UvCCHC4-1* vs. HWD-2. (**B**) Heatmap of FRKM-normalized transcript levels of the 810 DEGs in each pair-wise comparison. (**C**) GO enrichment analysis of the DEGs. MF: Molecular function; BP: Biological process; CC: Cellular component. (**D**) KEGG enrichment analysis of the DEGs. (**E**) RT-qPCR to validate the DEGs. The relative transcript abundance of each gene from Δ*UvCCHC4-1* was normalized by comparison with the gene from HWD-2 (relative transcript level = 1). The asterisks represent significant differences between Δ*UvCCHC4-1* and HWD-2 at *p* = 0.05.

To investigate the regulatory mechanism of the transcription factor UvCCHC4, we performed RNA-seq analysis to compare the gene expression profiles of HWD-2 vs. the ∆*UvCCHC4-1* mutant using infected spikelets at 3 dpi. Based on log_2_(∆*UvCCHC4-1*/HWD-2) values > two-fold change, 810 differentially expressed genes (DEGs) were identified, including 652 downregulated and 158 upregulated genes (Figure 8A,B; Supplementary Table S2). Many of the DEGs were enriched in the GO terms structural constituent of ribosome, translation, ribosome, nucleolus, and rRNA binding (Figure 8C). KEGG enrichment analysis revealed that many DEGs were enriched in the pathways of mitochondrial biogenesis, ribosome, transporters, and ribosome biogenesis (Figure 8D). To confirm the gene expression patterns revealed by RNA-seq, we performed RT-qPCR analysis using ten selected genes. The expression pattern of each downregulated or upregulated gene was consistent with that in the RNA-seq data (Figure 8E). These results indicate that *UvCCHC4* plays key roles in ribosome activity and translation.

### 3.10. Subcellular Localization of UvCCHC4 and UvCCHC5 in U. virens

**Figure 9:**
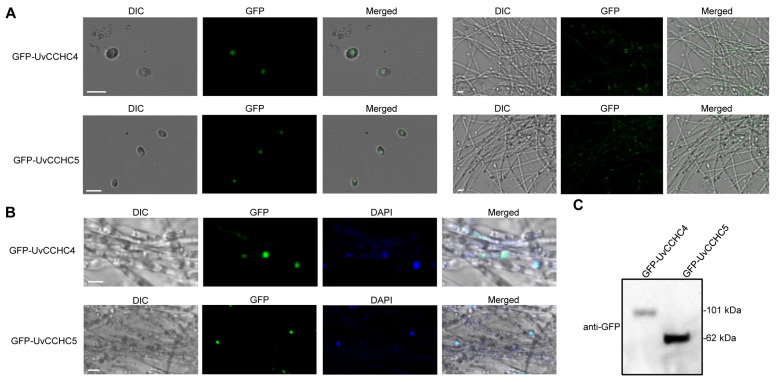
Subcellular localization of UvCCHC4 and UvCCHC5 in *U. virens*. (**A**) Subcellular localization of GFP-UvCCHC4 and GFP-UvCCHC5 in mycelia and conidia. DIC, differential interference contrast; GFP, green fluorescent protein; Scale bar = 10 μm. (**B**) Co-localization of DAPI and GFP-UvCCHC4 or GFP-UvCCHC5 in mycelia. DAPI, 4,6-diamidino-2-phenylindole. Scale bar = 10 μm. (**C**) Immunoblot analysis examining GFP-UvCCHC4 and GFP-UvCCHC5 expression using an anti-GFP antibody.

Finally, to explore the subcellular localizations of UvCCHC4 and UvCCHC5, we inserted the pNeo3300III-GFP-*UvCCHC4* and pNeo3300III-GFP-*UvCCHC5* constructs into *A. tumefaciens* strain EHA105 and transformed conidia of Δ*UvCCHC4-1* and Δ*UvCCHC5-23* with these two vectors, respectively. Under confocal microscopy, the GFP signals in the transformed strains were observed in the nuclei of both conidia and vegetative hyphae (Figure 9A,B). Immunoblotting of these transformed strains using anti-GFP antibody showed a 101-kDa GFP-UvCCHC4 band and a 62-kDa GFP-UvCCHC5 band, indicating that the GFP-UvCCHC4 and GFP-UvCCHC5 fusion proteins were not completely cleaved (Figure 9C). These results indicate that UvCCHC4 and UvCCHC5 are localized to the nucleus in *U. virens*.

## 4. Discussion

Rice false smut, caused by *U. virens*, is one of the most serious rice diseases worldwide [28]. Many studies on *U. virens* have focused on its infection processes and strategies, whereas few have focused on genes involved in the virulence of *U. virens*. The identification of *U. virens* virulence factors has been hindered by its slow mycelial growth rate, its infection organ specificity, time- and labor-consuming artificial inoculation methods, and the relative difficulty of genetic transformation. Our understanding of the molecular mechanisms underlying *U. virens* virulence is quite limited. However, the efficiency of gene replacement in *U. virens* increased when the CRISPR (clustered regularly interspaced short palindromic repeat)/Cas9 system was utilized to generate gene-knockout strains [45]. This led to the functional identification of various pathogenic genes in *U. virens*, including i*Uvt3277*, *UvCom1*, *UvPRO1*, *UvAc1*, *UvPdeH*, *SCRE1*, *SCRE2* (*UV_1261*), *UvBI-1*, *UvPmk1*, *UvCDC2*, *UvCdc3*, *UvCdc10*, *UvCdc11*, *UvCdc12*, *UvPal1*, *UvHox2*, *UvRpd3*, *UvSlt2*, *UvATG8*, *UvZnFTF1*, *UvMSN2*, *UvCGBP1*, and *UvEC1*. These genes play important roles in the hyphal growth, conidiation, stress responses, and virulence of *U. virens* [46,47,48,49,50,51,52,53,54,55]. In the current study, we identified and functionally characterized CCHC-type zinc-finger proteins in *U. virens*. The CCHC-type zinc-finger protein homologs are well conserved in other filamentous ascomycetes, such as *Magnaporthe oryzae* and *Fusarium graminearum*. However, none of these homologs in fungi have been functionally characterized. In this study, we determined that the deletion of *UvCCHC* genes caused defects in the hyphal growth, conidiation, stress responses, and virulence of *U. virens*.

The Δ*UvCCHC5* and Δ*UvCom1* mutants have the same infection phenotypes: Both mutants can infect the floral organs of rice, but the infected hyphae cannot expand to form smut balls. We propose that this defect is due to a problem in the nutrient supply chain established between the mutant fungus and rice. This defect renders the mutants incapable of utilizing large quantities of the nutrients produced by rice, preventing them from successfully forming smut balls. Previous studies have shown that deletion of *UvCom1* does not affect the expression of genes related to rice filling, but the Δ*UvCom1* mutant has defects in transmembrane transport, transporter activity, carbohydrate transport, and metabolism [35]. Here we showed that the Δ*UvCCHC5* and Δ*UvCom1* mutants induced the expression of rice-filling genes such as the wild type, whereas some transmembrane transport genes were significantly downregulated in the mutant fungi.

Biotrophic phytopathogenic fungi infect plants to obtain nutrients by redirecting carbon and nitrogen resources. Major facilitator superfamily (MFS) transporters target a wide range of substrates, including lipids, ions, amino acids and peptides, carbohydrates, and nucleosides [56]. Various genes related to MFS and transmembrane transport were significantly downregulated in mutant mycelia and during *U. virens* infection, including *Uv8b_6013* (MFS multidrug transporter), *Uv8b_6977* (Sugar transporter family protein), *Uv8b_6685* (MFS multidrug transporter), *Uv8b_144* (Carboxylic acid transport protein), *Uv8b_6649* (MFS transporter), *Uv8b_7122* (MFS sugar transporter), and *Uv8b_827* (Oligopeptide transporter). UvCCHC5 and UvCom1 are two key transcription factors: further analysis of their activities could enrich our understanding of the formation of smut balls and the interaction between rice and *U. virens*. Whether the downstream target genes of the transcription factors UvCCHC5 and UvCom1 are the same, and whether they regulate the same pathway, will require further study.

We also performed RNA-seq to identify DEGs between HWD-2 and Δ*UvCCHC4-1*. Bioinformatics analysis revealed that several of these DEGs are enriched in the categories of structural constituent of ribosomes, translation, ribosome, nucleolus, rRNA binding, mitochondrial and ribosome biogenesis, and transporters. To further explore whether the genes regulated by *UvCCHC4* are involved in the interaction between *U. virens* and rice, we searched the PHI-base database to identify DEGs regulated by *UvCCHC4*, including genes encoding different types of transcription factors and transporters, G-protein-coupled receptors, protein kinases, and putative effectors. The results suggest that *UvCCHC4* regulates diverse processes to mediate the interaction between *U. virens* and rice. Many DEGs encode secreted proteins (Supplementary Figure S3), including *Uv8b_44*, *Uv8b_2253*, *Uv8b_2286*, *Uv8b_5436*, *Uv8b_5518*, and *Uv8b_6470*, which were previously reported to induce cell death in *Nicotiana benthamiana* and rice [31,57]. We propose that UvCCHC4 helps regulate the expression of secreted proteins, which may themselves play important roles in the pathogenic process of *U. virens*. The roles of these secreted proteins in pathogenicity should be investigated in the future.

In summary, this is the first study to uncover the roles of CCHC-type zinc-finger proteins in regulating the development and virulence of a phytopathogen. Our findings shed light on the regulatory mechanism underlying the formation of smut balls.

## 5. Conclusions

In summary, our study revealed that *UvCCHC* genes affected the stress responses, vegetative growth, conidiation, and virulence of *U. virens*. We identified an important transcription factor, *UvCCHC5*, from this pathogen. *UvCCHC5* was essential for the formation of rice smut balls; *∆UvCCHC5* mutants infected rice spikelets normally, but could not form smut balls, and lost the ability to stably utilize nutrients from the rice host. Sugar utilization experiments showed that the ∆*UvCCHC5* mutants were defective in the utilization of glucose, sucrose, lactose, stachyose, and trehalose. ∆*UvCCHC5* mutants induced the expression of rice-filling genes such as the wild type, whereas some transmembrane transport genes were significantly downregulated in the mutant fungi, and the resulting nutrient deficiencies prevent them from utilizing nutrients from rice to form smut balls. These findings improve our understanding of the molecular mechanism underlying the formation of smut balls in rice by *U. virens*.

## Figures and Tables

**Table 1:** Genome-wide identification of CCHC-type zinc-finger genes in *U. virens*.

Gene ID	Function Description	Name
Uv8b_1580	Pre-mRNA-splicing factor slu7	*UvCCHC1*
Uv8b_2941	Retinoblastoma-binding protein	*UvCCHC2*
Uv8b_4070	Cellular nucleic acid-binding protein	*UvCCHC3*
Uv8b_4168	Zinc-knuckle domain-containing protein	*UvCCHC4*
Uv8b_5736	Putative zinc-knuckle transcription factor	*UvCCHC5*
Uv8b_5874	Zinc-finger domain-containing protein	*UvCCHC6*
Uv8b_8085	Zinc-knuckle domain-containing protein	*UvCCHC7*

**Table 2:** Wild-type and mutant strains of *U. virens* used in this study.

Strain	Description	Reference
HWD-2	Wild-type strain	[35]
Δ*UvCCHC1-5*	*UvCCHC1* deletion mutant of HWD-2	This study
Δ*UvCCHC1-73*	*UvCCHC1* deletion mutant of HWD-2	This study
CΔ*UvCCHC1-5*	*UvCCHC1* complementation strain from Δ*UvCCHC1-5*	This study
Δ*UvCCHC2-36*	*UvCCHC2* deletion mutant of HWD-2	This study
Δ*UvCCHC2-51*	*UvCCHC2* deletion mutant of HWD-2	This study
CΔ*UvCCHC2-36*	*UvCCHC3* complementation strain from Δ*UvCCHC2-36*	This study
Δ*UvCCHC3-16*	*UvCCHC3* deletion mutant of HWD-2	This study
Δ*UvCCHC3-37*	*UvCCHC3* deletion mutant of HWD-2	This study
CΔ*UvCCHC3-16*	*UvCCHC3* complementation strain from Δ*UvCCHC3-16*	This study
Δ*UvCCHC4-1*	*UvCCHC4* deletion mutant of HWD-2	This study
Δ*UvCCHC4-11*	*UvCCHC4* deletion mutant of HWD-2	This study
CΔ*UvCCHC4-1*	*UvCCHC4* complementation strain from Δ*UvCCHC4-1*	This study
Δ*UvCCHC5-23*	*UvCCHC5* deletion mutant of HWD-2	This study
Δ*UvCCHC5-43*	*UvCCHC5* deletion mutant of HWD-2	This study
CΔ*UvCCHC5-23*	*UvCCHC5* complementation strain from Δ*UvCCHC5-23*	This study
Δ*UvCCHC6-8*	*UvCCHC6* deletion mutant of HWD-2	This study
Δ*UvCCHC6-22*	*UvCCHC6* deletion mutant of HWD-2	This study
CΔ*UvCCHC6-8*	*UvCCHC6* complementation strain from Δ*UvCCHC6-8*	This study
Δ*UvCCHC7-18*	*UvCCHC7* deletion mutant of HWD-2	This study
Δ*UvCCHC7-63*	*UvCCHC7* deletion mutant of HWD-2	This study
CΔ*UvCCHC7-18*	*UvCCHC7* complementation strain from Δ*UvCCHC7-18*	This study

## Supplementary Materials

The following are available online at https://www.mdpi.com/article/10.3390/jof7110947/s1. Figure S1: Deletion and complementation of *UvCCHC* genes in *U. virens*. (**A**) Deletion and complementation strategies for *UvCCHC* genes. (**B**) PCR verification of four *UvCCHC* deletion mutants using primer pairs P1/P2, P3/P4, P3/P6 and P5/P6. (**C**) RT-PCR analysis of HWD-2 and Δ*UvCCHC* mutant and complementation strains; Figure S2: (**A**–**G**) Colony growth of Δ*UvCCHC* mutant strains on PSA supplied with 0.25 M NaCl, 0.5 M sorbitol, 0.02% or 0.04% H_2_O_2_, 0.03% SDS, 0.12 mg/mL CR, or 0.12 mg/mL CFW after 14 d of incubation at 28 °C; Figure S3: Heatmap of the levels of secreted proteins in the ∆*UCCHC4-1* mutant and HWD-2; Table S1: PCR primer pairs used in this study; Table S2: DEGs in rice spikelets infected with HWD-2 and Δ*UvCCHC4-1* at 3 dpi.
