# Supplementary figures and images for: Genome-Wide Identification and Functional Characterization of CCHC-Type Zinc Finger Genes in Ustilaginoidea virens
Source: J Fungi (Basel). 2021 Nov 10;7(11):947. doi: 10.3390/jof7110947 (PMC8619310; doi:10.3390/jof7110947)

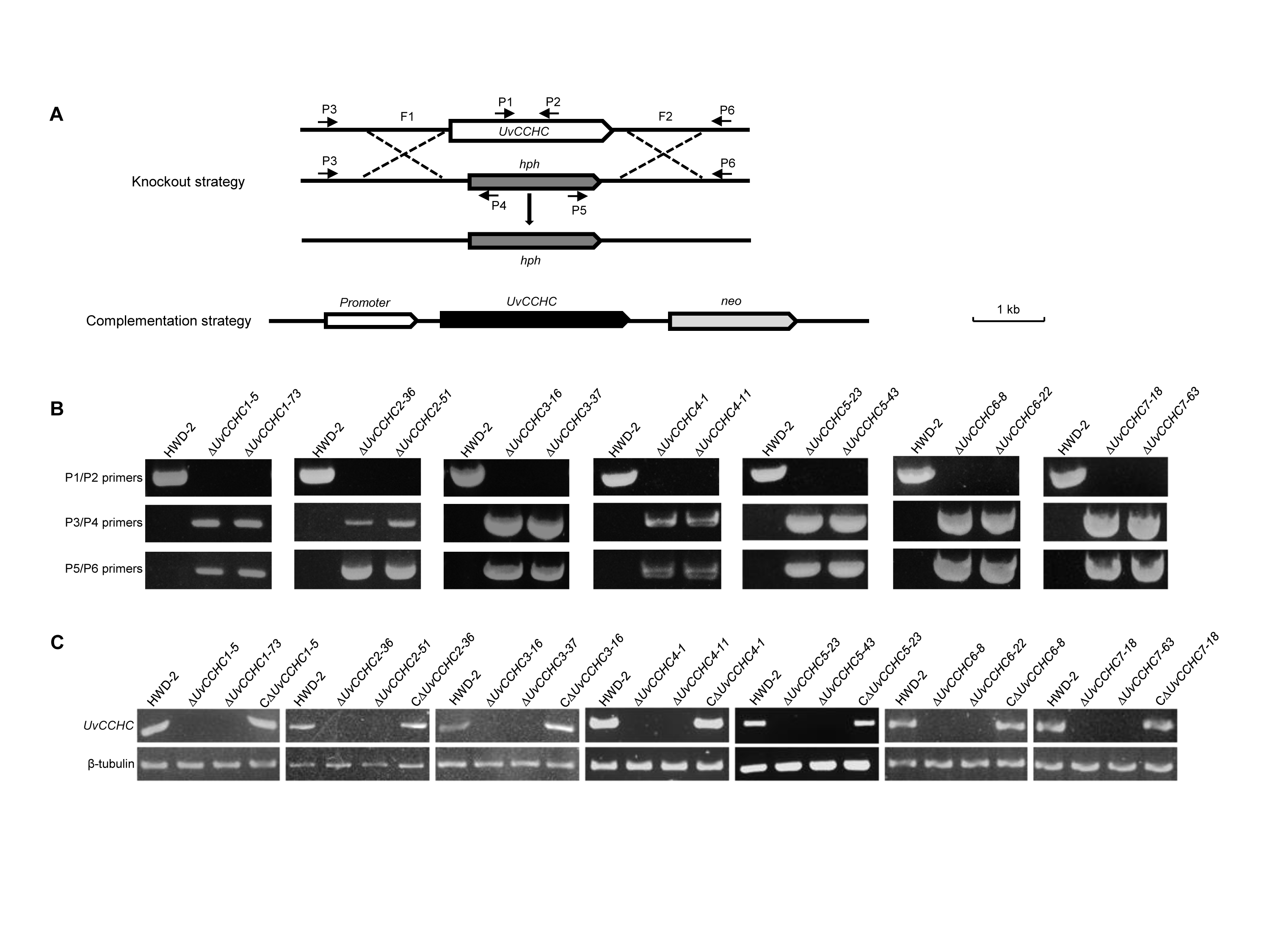

Supplement: Supplementary file 1 [file jof-07-00947-s001.zip › figure S1.tif]

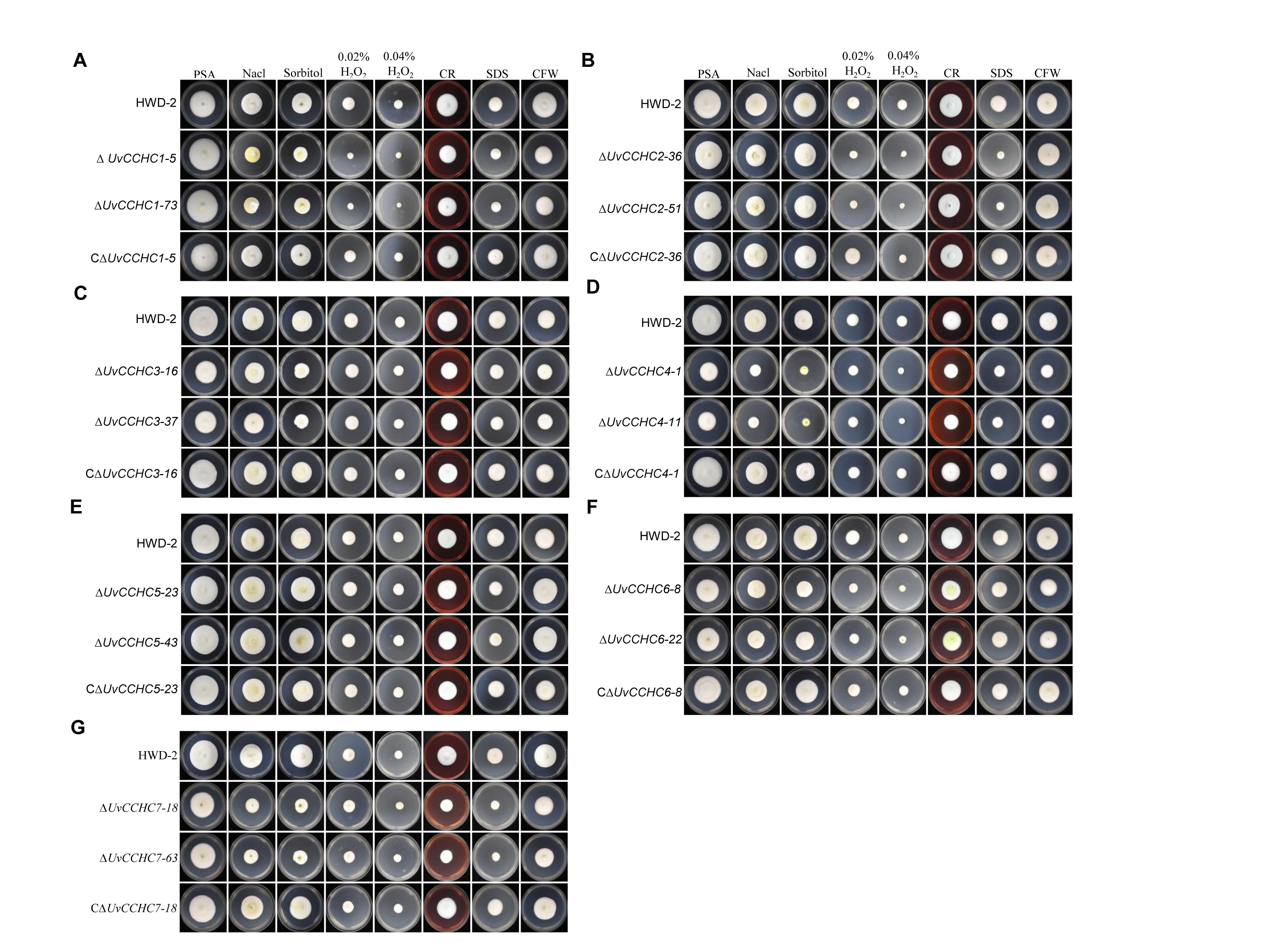

Supplement: Supplementary file 1 [file jof-07-00947-s001.zip › figure S2.tif]

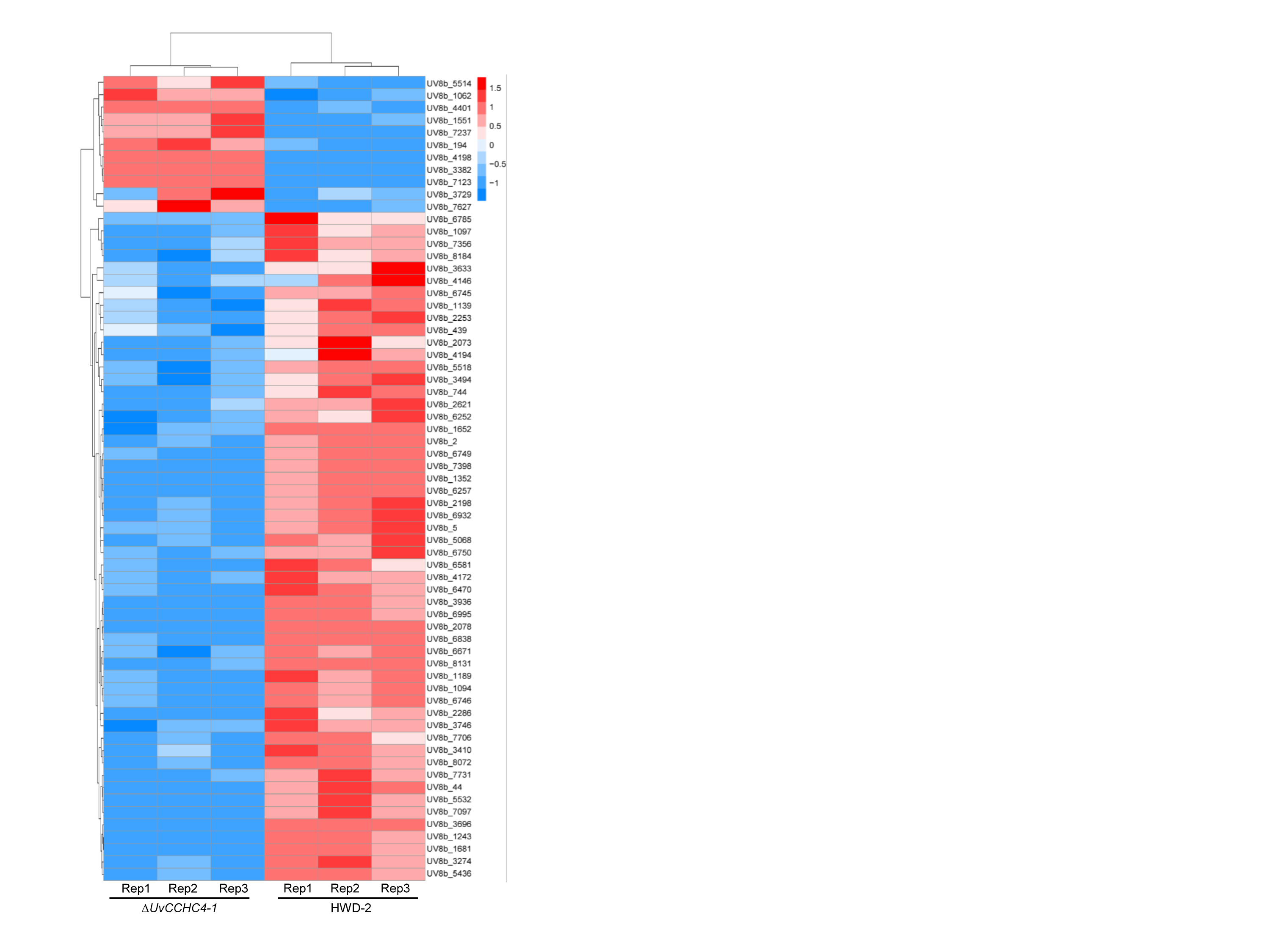

Supplement: Supplementary file 1 [file jof-07-00947-s001.zip › figure S3.tif]
